# Simultaneous Measurement of Cortisol, Cortisone, Dexamethasone and Additional Exogenous Corticosteroids by Rapid and Sensitive LC–MS/MS Analysis

**DOI:** 10.3390/molecules28010248

**Published:** 2022-12-28

**Authors:** Federico Ponzetto, Mirko Parasiliti-Caprino, Fabio Settanni, Antonello Nonnato, Giulio Mengozzi, Ezio Ghigo, Roberta Giordano

**Affiliations:** 1 Division of Endocrinology, Diabetes and Metabolism, Department of Medical Sciences, University of Turin, 10126 Turin, Italy; 2 Clinical Biochemistry Laboratory, City of Health and Science University Hospital, 10126 Turin, Italy; 3 Department of Clinical and Biological Sciences, University of Turin, 10126 Turin, Italy

**Keywords:** LC–MS/MS, steroids, serum, cortisol, cortisone, dexamethasone, hypercortisolism, Cushing’s syndrome, diagnosis

## Abstract

The simultaneous measurement of dexamethasone and cortisol has proven the ability to increase the diagnostic performance of the overnight dexamethasone-suppression test. Furthermore, the therapeutic drug monitoring of administered corticosteroid drugs could represent a crucial tool for investigating unexpected variations of steroid hormones’ circulating levels. In this work, an LC–MS/MS method for the quantification of cortisol, cortisone, dexamethasone and six additional exogenous corticosteroids in the serum/plasma matrix was developed and validated in compliance with the ISO/IEC requirements. To assess the efficiency of the validated method, serum samples of 75 patients undergoing the dexamethasone-suppression test and 21 plasma samples of patients under immunosuppressive treatment after kidney transplant were analyzed. In all dexamethasone-suppression test samples, it was possible to measure the circulating levels of cortisol, cortisone and dexamethasone. Concentrations of the latter were for all tested patients above the proposed cutoff for the dexamethasone-suppression test’s results, and the cortisol concentrations showed good correlation with the ones measured by routine immunometric analysis, therefore confirming the screening outcome for all enrolled patients. Prednisone was detected and quantified in all enrolled patients, confirming the use of such a corticosteroid for immunosuppressive therapy. Thanks to these two applications, we proved the overall performance of the developed LC–MS/MS method for four target analytes. The future implementation of such an analytical tool in the clinical biochemistry laboratory’s routine will guarantee a single and versatile tool for simultaneously monitoring dexamethasone-suppression-test results and corticosteroid drugs’ administration.

## 1. Introduction

Dexamethasone (Dex) is an exogenous fluorinated glucocorticoid drug that is used for its anti-inflammatory properties in the treatment of many pathological conditions [1,2]. Thanks to its ability to challenge the hypothalamic–pituitary–adrenal (HPA) axis, hence temporarily suppressing cortisol (F) production, and besides its therapeutic use, it has also been employed in the 1 mg dexamethasone-suppression test (DST) and in the low-dose 2-day dexamethasone test (LDDT). Based on clinical suspicion [3], these tests are recommended first-level investigations for the diagnosis of Cushing’s syndrome (CS) [4,5], and DST is the key tool for diagnosing autonomous cortisol secretion (ACS) in patients with adrenal incidentaloma [6]. According to international protocols for the DST, the patient is asked to take 1 mg of Dex at 11:00 p.m., and a blood sampling is performed the following morning, between 8:00 and 9:00 a.m. for the F-serum-concentration measurement. Meanwhile, for LDDT, the patient is asked to take 0.5 mg of Dex every 6 h for 48 h, and a blood sampling for F-serum concentration is performed the morning of the third day, between 8:00 and 9:00 a.m. When exploring the suspicion of CS, F-concentration values after tests higher than the cutoff of 18 ng/mL (50 nmol/L) should be interpreted as positive [6]. Furthermore, when a DST is performed on patients with adrenal incidentaloma and without clinical signs of CS, ACS diagnosis could be assigned when insufficient F suppression is observed after DST: ACS is suggested when measured F concentration is above 50 ng/mL (138 nmol/L), while for values between 18 and 50 ng/mL, “possible ACS” could be envisaged [7,8]. However, positive DST and LDDT outcomes should lead to further diagnostic investigations by late-night salivary cortisol or 24-hour urine-free cortisol measurements [9,10,11,12]. Although the application of the 18 ng/mL as cutoff value for the DST and LDDT screening could achieve 95% of sensitivity and 80% specificity for CS diagnosis [4], false-positive results could lead to additional follow-up examinations that are a matter of concern for both the healthcare system and the patients. The lack of specificity of the dexamethasone-suppression tests is related to the variation of Dex circulating levels following its oral administrations [13,14,15] due to several factors: variable gastrointestinal drug absorption, interindividual differences in metabolism, food or drug interactions, inactivation by conversion by CYP3A4 in the liver and renal clearance [16,17]. Moreover, the missed Dex ingestion by the patient is often suspected but is not possible to be proven by routinely performed assays. For all of these reasons, the interest in the simultaneous quantification of F and Dex circulating concentrations is constantly growing, as it represents a valuable analytical tool for obtaining more reliable test results [18,19]. To obtain precise and accurate measurements of F and Dex in serum/plasma matrix, liquid chromatography/tandem mass spectrometry (LC–MS/MS) techniques are currently the gold-standard assays, improving selectivity, sensitivity, analysis’ speed and throughput in comparison with previously employed immunoassays.

In the last two decades, several research groups have developed in-house validated LC–MS/MS methods for the quantification of synthetic glucocorticoids in serum for clinical applications. Most of published research works dealing with therapeutic drug monitoring and pharmacokinetic studies describe LC–MS/MS methods aiming at the monitoring of a single exogenous compound (e.g., betamethasone, beclomethasone dipropionate, budesonide, prednisone and prednisolone), eventually also measuring within the same analysis relative target metabolites and/or circulating F [20,21,22,23]. A few examples of extended panels were also described: Taylor et al. [24] set up a 15 min LC–MS/MS method for the detection and quantification of 15 exogenous corticoids (including Dex) plus F to be applied to both serum and urine samples, as well as to tablet extracts; and Methlie et al. [25] developed a sensitive method that is capable of measuring serum concentrations of Dex, prednisone and prednisolone together with a small panel of clinically relevant steroid hormones. By evaluating the analytical conditions employed in the literature, it is possible to observe that different sample preparation protocols have been employed to extract steroid analytes from biological fluids, with protein precipitation (PPE) and liquid–liquid extraction (LLE) being the most used techniques, followed by solid-phase extraction (SPE) and more recently by supported liquid extraction (SLE). Regarding analytes’ separation, reversed-phase chromatography represents the gold standard, and it has been used equipping chromatographic systems with C18 LC columns of different dimensions. A brief summary of analytical conditions and performance of most relevant research works describing the LC–MS/MS method and including F, E and Dex is presented in Table 1.

Thanks to such technology, recent studies were able to investigate the possibility of increasing the specificity of the DST and LDDT by monitoring Dex concentrations and establishing method-specific cutoff values of 2.2 ng/mL (5.6 nmol/L) and 1.3 ng/mL (3.3 nmol/L) for immunoassay and LC–MS/MS analysis, respectively [18,29]. The aim of the present study was to develop and validate a rapid and sensitive LC–MS/MS method for the simultaneous determination of Dex and F in a single-run analysis of the same serum or plasma sample. Furthermore, the monitoring of the circulating levels of most commonly administered exogenous corticosteroids represents a strategic tool for investigating unexplained F suppression in endocrinological patients [30]. With the aim of providing clinical biochemistry laboratories with a more comprehensive and versatile analytical tool, we also included in the target analytes’ list of the LC–MS/MS method cortisone (E) and an additional six corticosteroid drugs that are routinely used for therapeutic purposes and that could provoke the perturbation of the HPA axis, as well as of the whole steroidogenesis pathway.

## 2. Results and Discussion

### 2.1. Method Development

**Figure 1:**
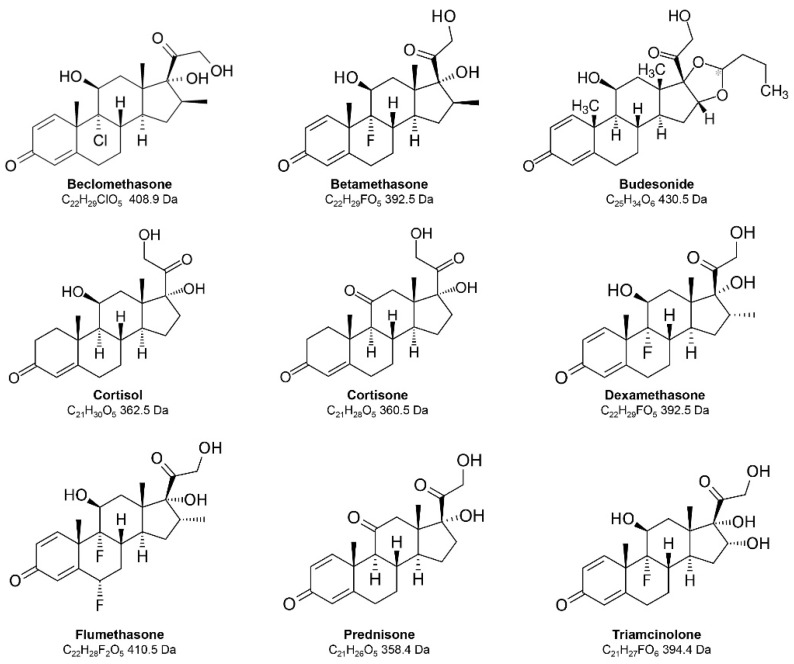
Chemical structure of target analytes.

The aim of the present study was the development of a rapid and sensitive LC–MS/MS method for the simultaneous measurement of F, E and seven exogenous corticosteroids in human serum. The first aspect that was studied during method development was the chromatographic separation of the nine target analytes that share the classical cyclopentaphenanthrene skeleton of steroidal compounds but present a large variety of substituents, including, in the case of exogenous compounds, the presence of fluorine and chlorine in Position 9 (chemical structures in Figure 1).

**Figure 2:**
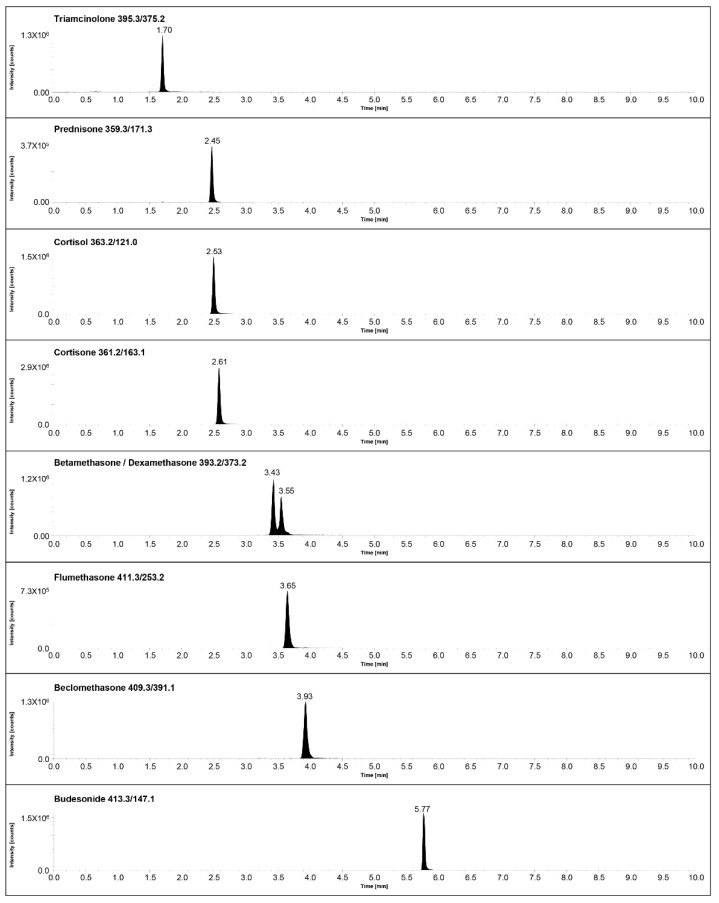
Chromatograms obtained with the optimized chromatographic conditions for a solution containing all target analytes in MeOH/H_2_O (1:1, *v*/*v*) at a concentration of 10 ng/mL.

Deionized water and MeOH with the addition of 0.1% FA were initially employed as LC mobile phases, and, with the aim of investigating the retention of target analytes, a first generic linear gradient from 10% to 90% B in 10 min was tested. Thanks to this first experiment, it was possible to observe that all target compounds were eluted at a low concentration of organic solvent (<45%), with the exception of Bud, and therefore it was decided to divide the gradient in two different steps with appropriate slopes. If the separation of Bud did not represent a critical challenge during development, the separation of the other eight analytes required an isocratic step to be achieved. Although employing a first isocratic step of the gradient improved the separation of early eluting compounds, using MeOH as organic solvent, it was not possible to efficiently separate at baseline the two isomers Bet and Dex. To overcome this issue, it was decided to replace MeOH with ACN, and after having adjusted the percentage of organic solvent in the isocratic step, the baseline separation of challenging isomers was achieved. Concerning the elution of Bud, a different type of issue was raised: indeed, due to the presence of 22R and 22S epimers in both certified reference materials and pharmaceutical preparations [31], with the optimized chromatographic separation, a double peak for Bud was initially obtained. With the purpose of facilitating Bud peak integration and, consequently, its quantification in biological samples, it was decided to increase the slope of the gradient, replacing the initially optimized second step of the gradient, which was passing from 35% to 98% B in 5 min, with a faster one, passing from 65% to 98% B in 1.5 min, obtaining a unique peak for Bud with the coelution of both its epimers. The chromatographic peaks obtained with the initial and the optimized gradients are presented in Supplementary Materials Figure S1. Once we obtained a satisfactory chromatographic separation of all endogenous and exogenous steroids, a sensitivity issue was encountered. In fact, the use of 0.1% FA as a mobile-phase modifier was not sufficient to increase the analytes’ ionization and to reach optimal sensitivity levels for the detection of target compounds in human serum. Therefore, it was decided to use NH_4_F as an aqueous mobile-phase modifier since it has proven its ability to enhance steroid ionization in LC–MS/MS applications, with a particular focus on most challenging analytes [32,33,34]. The final chromatographic conditions, as detailed in Section 3.3, allowed for the separation of target analytes with a satisfactory sensitivity, and an example of chromatograms obtained injecting a solution containing all the analytes standards at a concentration of 10 ng/mL in MeOH/H_2_O (1:1, *v*/*v*) is presented in Figure 2. The second part of the method development focused on the optimization of a simple and robust sample-preparation protocol that is capable of efficiently extracting the steroidal compounds without causing significant matrix effects. For this aim, the SLE format, which guarantees excellent results with steroidal analytes [35,36,37], was chosen, and experiments employing three different organic solvents in the elution step were performed. The extraction recoveries and matrix effects, calculated as described in Section 3.4 with Matuszevsky’s approach [38], were assessed by using DCM, EtAc and TBME as elution solvents.

**Figure 3:**
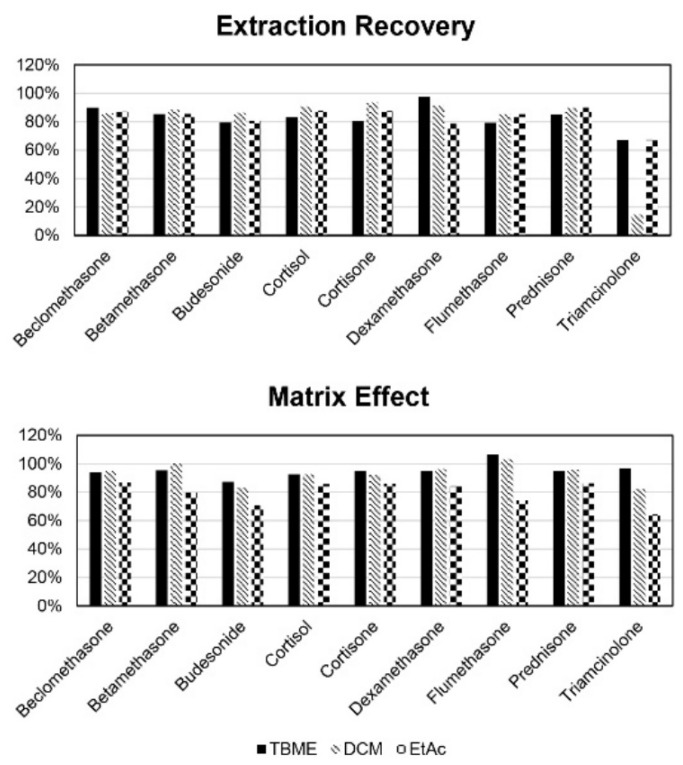
Summary of extraction recoveries and matrix effects obtained for target analytes by employing TBME, DCM and EtAC as elution solvents in the SLE sample-preparation procedure.

The obtained results are summarized in Figure 3 and clearly highlight a poor extracting performance for Tri when DCM was used for eluting the steroids from the SLE plate. On the other hand, the performances obtained with TBME and EtAC were similar, with the latter showing slightly lower recovery values. In detail, extraction recoveries ranging from 67.0% (Tri) to 97.8% (Dex) were obtained with TBME and from 67.3% (Tri) to 90.0% (Pred) with EtAc. Regarding the matrix effects, as it is possible to observe in Figure 3, the best performance was achieved by employing TBME, followed by DCM, while the worst performance was obtained by using EtAc. In particular, matrix effects ranging from 87.3% (Bud) to 106.6% (Flu) were observed with TBME, from 82.4% (Tri) to 103.2% (Flu) with DCM and from 64.2% (Tri) to 86.6% (Bec) with EtAc. Taking into account the outcomes of these experiments, TBME was chosen as the elution organic solvent, and the final sample preparation procedure (described in Section 3.2) was employed during the study.

### 2.2. Method Validation

To validate the developed LC–MS/MS method, a dedicated quantitative validation experimental protocol was set up to be compliant with the ISO/IEC requirements [39]. The first parameter that was investigated was the method’s selectivity. For this purpose, the development of an effective and reproducible sample-preparation protocol that was based on SLE extraction, the optimization of chromatographic separation and the selection of two MS/MS transitions for each target analyte by direct infusion in the MS system guaranteed satisfactory selectivity for the method. Details regarding the retention times of each target analyte and the selected MS/MS transitions are presented in Table 2 and Table 3, respectively. To further verify the method’s selectivity, five depleted serum samples and five depleted serum samples spiked with a solution containing sixty-three endogenous steroid hormones at a concentration of 10 ng/mL (details in Supplementary Materials Table S1) were extracted and analyzed. When evaluating the extracted ion chromatograms of all target analytes’ MS/MS transitions in the elution region of each analyte, no significant differences were observed while comparing the negative samples with the ones spiked with 63 endogenous steroid hormones; therefore, the absence of potential interferences (<20% LLOQ) was assessed, and the satisfactory selectivity of the developed method was demonstrated. The performance of the sample-preparation protocol in terms of extraction recovery and matrix effect was assessed by employing the methodology proposed by Matuszevsky et al. [38]. Thanks to the optimized SLE procedure, satisfactory extraction recoveries comprised between 79.2% (Flu) and 97.8% (Dex), and negligible matrix effects ranging from 87.3 (Bud) to 106.6% (Flu) were obtained. An exception in extraction efficiency was observed in the case of Tri, for which the poorest recovery was measured (67.0%). This experimental result, which was already highlighted during method development, can be explained by the fact that Tri is the compound showing the highest hydrophilicity among the target analytes (is also the first analyte eluted from the C18 LC column in reversed phase chromatography) and that the SLE extraction efficiency is directly proportional to the hydrophobicity of extracted analytes. However, the obtained recovery was judged satisfactory for the application of the LC–MS/MS method in clinical context, providing an excellent quantitative performance down to 1 ng/mL concentration in serum/plasma. The analysis of three extracted negative controls immediately after the most concentrated calibration sample (Level 6) allowed us to investigate the presence of potential carryover. The measured analytes’ peak areas in negative serum samples ranged from 0.2% (Dex) to 0.6% (F) of the one measured in the Level 6 calibration sample, hence highlighting the negligible carryover for all target analytes.

The quantitative validation protocol was performed in three analytical series by three different operators, analyzing the six levels of the calibration curve in duplicate, as well as the six levels of validation samples in quadruplicate, and it assessed the satisfactory trueness and precision values for all target analytes. Calibration curves were prepared by spiking charcoal–dextran-stripped serum at six different concentrations for each analyte obtaining determination coefficient above 0.99 for all compounds, hence highlighting the satisfactory linearity of the method in the investigated ranges (calibration lines’ equation in Supplementary Materials Table S2). As it is possible to observe in Table 2, where a summary of all quantitative validation results, along with target analytes’ retention times and measured extraction recoveries and matrix effects, is presented, the developed method showed satisfactory values for trueness, repeatability, intermediate precision and combined uncertainty. In more detail, trueness (explaining method’s accuracy) ranging from 93.2% and 108.4% and repeatability (explaining method’s precision) comprising between 4.6% and 10.3% were measured during the validation protocol, highlighting the satisfactory quantitative performance of the method characterized by deviations in terms of precision and accuracy lower than 15%. Furthermore, the combined uncertainty was also obtained by quadratic combinations of the intermediate precision and the root mean square of the bias estimates for each of the six investigated concentration levels, showing values that ranged from 7.4% to 14.1%, hence being for all target analytes at all monitored concentration levels below the threshold acceptance value of 20%, as defined in the quantitative validation protocol. The lowest concentration for which a combined uncertainty lower than 20% was measured was considered to be the LLOQ, and for all compounds included in the developed method, this coincided with the lowest concentration of the prepared calibration/validation samples. The LLOQ values obtained for F (1 ng/mL) and E (0.1 ng/mL) allowed for the precise and accurate measurement of their circulating levels also in samples after DST with a negative clinical outcome (correct suppression of the HPA axis), thus improving the analytical performance on the routinely employed immunoassay that owns an LLOQ value for F of 10 ng/mL. Regarding the exogenous corticosteroids, the obtained LLOQ values were comprised between 250 pg/mL (Pred) and 1 ng/mL (Flu, Tri), which are considered suitable for detecting such analytes in patients’ serum samples [24]. The method’s robustness was assessed during extractions for quantitative validation, employing two different operators across three different days of quantitative validation; different mobile phases and solutions for SLE extraction were prepared for each day; analytical LC columns of two different lots were used; and, in addition, instrument maintenance (ESI source cleaning) was performed before each analytical batch. With all of these variations, the calibration lines (presented in Supplementary Materials Table S2) were satisfactory with an R^2^ greater than 0.98, and the measurement of uncertainty performed gave values lower than 20% for all target analytes’ compounds. Therefore, the developed method was considered robust in the range of linearity for each compound. Finally, the stability of the extracted samples was assessed by storing at 4 °C four replicates of external quality controls (QCs), containing F, E and Dex at high concentrations, as well as four replicates of Level 5 calibration samples analyzed at Day 0 and reinjecting them three (Day 3) and seven (Day 7) days later. The concentrations of target analytes measured at Day 0 were compared to those obtained from extracts stored for three and seven days at 4 °C. The quantification results are presented in Supplementary Materials Table S3 and show concentration differences lower than 15% for all target analytes. These results proved that the extracted compounds are stable in collection plates for at least seven days at 4 °C, highlighting useful information for the application of the described method in clinical biochemistry laboratories’ routine. A summary of the obtained satisfactory results of the validation protocol, detailing the acceptance criteria adopted for all investigated parameters, as well as the results obtained for each target analyte, is presented in Supplementary Materials Table S4.

### 2.3. Real Samples’ Applications

The performance of the developed and validated LC–MS/MS method were tested by analyzing two different sets of real biological samples coming from two different clinical studies carried out at the City of Health and Science University Hospital of Turin. Thanks to the first set of samples, which were collected from patients with suspected hypercortisolism performing DST or LDDT, it was possible to assess the reliability of the method regarding the measurement of F-, E- and Dex-serum concentrations. The seventy-five serum samples collected during this study were analyzed in five different analytical batches, and with the aim of verifying the accuracy of the analytes’ measurement, in each analytical batch, an external QC containing F, E and Dex at low and high concentration levels (F, 9.5 ng/mL and 176.9 ng/mL; E, 2.4 ng/mL and 44.2 ng/mL; Dex, 2.4 ng/mL and 44.2 ng/mL; Steroids in Serum LC/MS kit, Tecan, Mannedorf, Switzerland) was also analyzed. In all the five batches of this study, the analytes’ concentrations measured in QC samples did not deviate from the nominal concentration by more than 15%, thus providing further evidence of the quantitative performance of the developed method.

**Figure 4:**
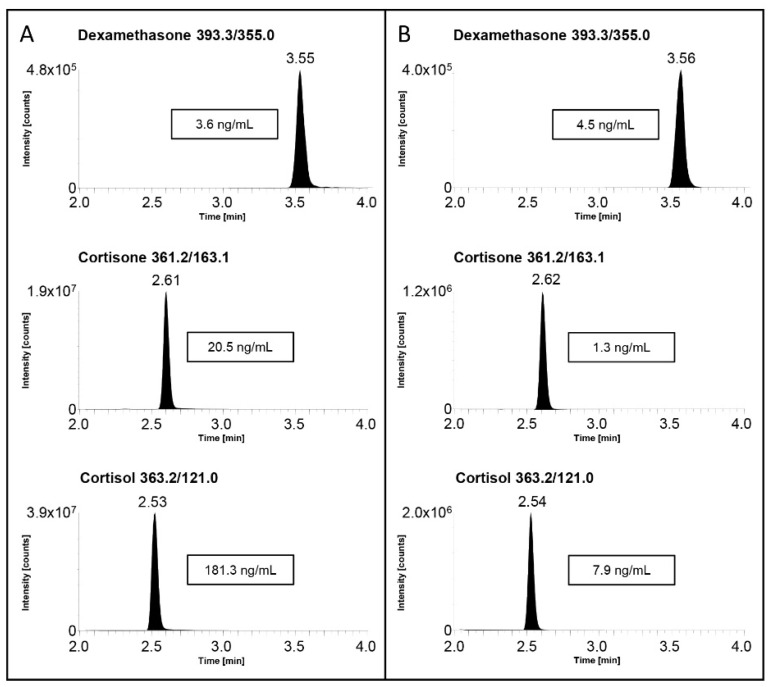
Examples of extracted ion chromatograms of Dex, E and F for (**A**) real sample of a patient not showing suppression after DST and (**B**) real sample of a patient with correct suppression after DST.

Based on the measured F-serum concentration after DST/LDDT and following the clinical guidelines [4,5], the patients were divided in two groups: one with the 21 patients showing positive test results ([F] > 18 ng/mL) and the other one with the 54 patients showing negative results ([F] < 18 ng/mL). The method was able to quantify F, E and Dex in all 75 samples included in both the abovementioned groups, and an example of chromatograms obtained from a positive and a negative DST sample was shown in Figure 4. The measured F-serum concentrations were in accordance with the one obtained with routine immunochemistry analysis, although the majority of analyzed samples (43 out of 75) were showing a concentration below immunoassay LLOQ of 10 ng/mL. The obtained Dex concentrations ranged from 1.4 ng/mL to 10.1 ng/mL in patients performing the DST and from 2.3 ng/mL to 17.1 ng/mL in patients performing the LDDT; hence, they correlated with the administered dosages. Furthermore, the LC–MS/MS analyses permitted us to quantify Dex-serum concentrations above the suggested threshold value of 1.3 ng/mL in all 75 samples [18], hence confirming the successful administration and metabolization of Dex and allowing the clinicians to accept the test results and proceed with patient management according to clinical practice.

**Figure 5:**
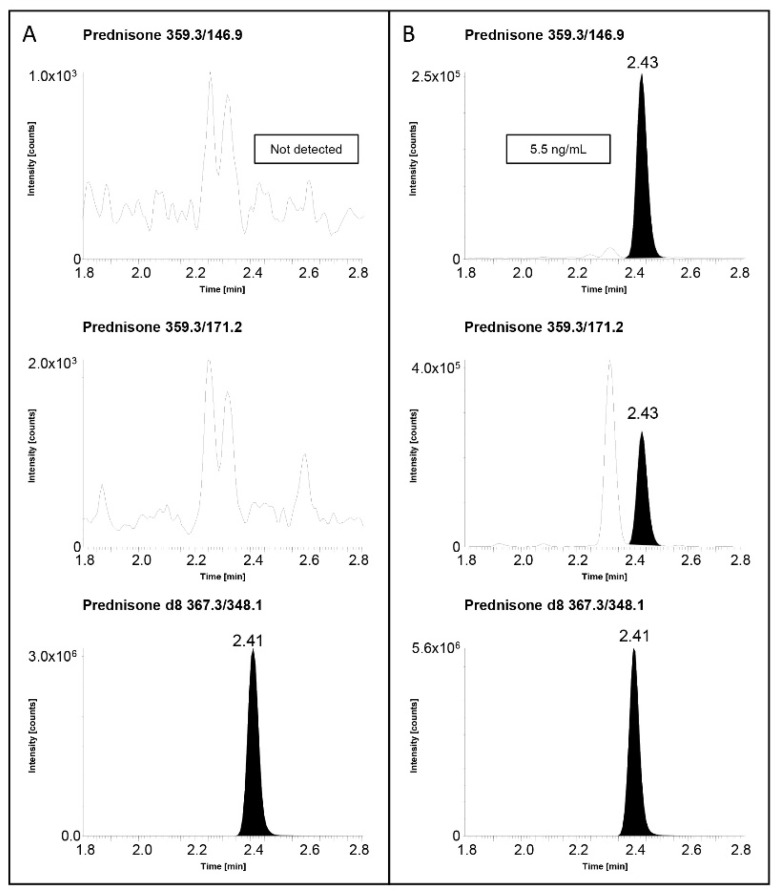
Examples of extracted ion chromatograms of the MRM transition of Pred and relative internal standards for (**A**) real sample of patient performing DST/LDDT not administered with prednisone and (**B**) real sample of a transplanted patient under treatment with prednisone.

The second set of samples, including plasma samples collected from patients who were subjected to kidney transplantation and currently under treatment with Pred for preventing organ rejection, allowed us to further assess the validated LC–MS/MS method’s performance in a real case scenario. The 21 plasma samples collected during the clinical study were analyzed in one analytical batch, but unfortunately, in the case of the Pred measurement, it was not possible to integrate into the analytical batch external QC samples, to have an additional test of the method’s accuracy. In all analyzed plasma samples, it was possible to detect Pred, with plasmatic concentrations ranging from 0.25 ng/mL to 5.5 ng/mL. As it is possible to observe in the chromatograms of Pred and relative internal standard’s MS/MS transitions presented in Figure 5, thanks to the developed method, it was possible to detect Pred in both monitored MS/MS transitions. Furthermore, a chromatogram obtained from the analysis of a DST/LDDT sample (hence not administered with Pred) was also shown in Figure 5 to demonstrate the absence of interfering signals in the elution region of Pred.

The results obtained from analyzing real serum samples proved the satisfactory analytical performance of the developed method. Nevertheless, the cost of analysis is an important aspect that has to be evaluated prior to the implementation of the presented LC–MS/MS method in the routine of clinical laboratories. For this purpose, we compared the cost per sample of the currently employed immunoassay for F measurement in serum, which is approximately EUR 7, with the one of the developed method, taking into account exclusively the employed consumables and considering 200 tests (DST + LDDST) performed in one year at the City of Health and Science University Hospital of Turin. The novel LC–MS/MS method has a cost of approximately EUR 10, which is 40% higher than the cost of the immunoassay. However, it has to be noted that the developed assay guarantees not only the measurement of F-serum concentrations, but also the accurate quantification of eight additional analytes, therefore creating a panel that could help in amortizing the costs’ increase. Furthermore, F-serum measurements currently account for more than 7000 tests in our laboratory (2022 data), and the introduction of the most expensive LC–MS/MS analysis for up to 200 requests, coming for all the Piedmont region (4 million inhabitants), would not have a significant impact on the laboratory’s annual budget. Last but not least, the implementation of the presented LC–MS/MS method should be considered only by laboratories with an already structured MS sector that could guarantee specialized personnel, which is a fundamental requirement for obtaining satisfactory results when dealing with MS-based analytical methods, and by hub reference hospitals that should centralize DST and LDDT requests coming from a wider region.

## 3. Materials and Methods

### 3.1. Chemicals and Reagents

Certified reference materials for endogenous steroid hormones, F and E, and labelled internal standards (IS), cortisol d4 and cortisone d8, were purchased from Steraloids (Newport, RI, USA). Reference standards for beclomethasone (Bec), betamethasone (Bet), budesonide (Bud), Dex, flumethasone (Flu), prednisone (Pred) and triamcinolone (Tri), as well as IS budesonide d8, dexamethasone d3 and budesonide d8, were provided by LGC Standards (Teddington, United Kingdom). UHPLC–MS-grade acetonitrile (ACN) was obtained from Carlo Erba Reagents S.r.l. (Cornaredo, Italy); UHPLC-grade formic acid (FA) was supplied by Biosolve BV (Valkenswaard, Netherlands); and ammonium fluoride (NH_4_F), tert-butyl methyl ether (TBME), dichloromethane (DCM) and ethyl acetate (EtAC) were provided by Merck KGaA (Darmstadt, Germany). Deionized water was obtained by a Milli-Q^®^-grade system (Millipore, Burlington, MA, USA) and was used for the preparation of all LC mobile phases and aqueous solutions. Charcoal–dextran-stripped human serum was acquired from Innovative Research Inc. (Novi, MI, USA). For each analyte and IS, stock solutions were prepared by dissolving 2 mg of powder in 2 mL of MeOH, obtaining a final concentration of 1 mg/mL; these stock solutions were stored in 2 mL amber glass vials at −80 °C until the preparation of intermediate solutions. The latter were prepared at appropriate concentrations (100 μg/mL, 10 μg/mL, 1 μg/mL and 100 ng/mL) by means of consecutive dilution of stock solutions in MeOH and finally stored in 10 mL glass tubes at −20 °C. Working solutions were prepared in MeOH containing all target analytes at different concentrations (concentration details in Supplementary Materials Table S5); such solutions were used for the preparation of calibration and validation samples by spiking 20 μL of the appropriate working solution in steroid-free serum. Furthermore, a mixture containing all IS (IS-mix) was prepared by spiking appropriate volumes of each IS intermediate in MeOH to reach the optimized concentration levels (details in Supplementary Materials Table S6). The IS-mix was stored in 10 mL glass tube at −20 °C until its use during sample preparation procedure. Fine optimization of IS concentration contained in the IS-mix was carried out, and finally the lowest concentration of IS detected in samples with a satisfactory repeatability without resulting in significant interference in analytes’ selected transitions was selected and used during validation and real samples’ analyses.

### 3.2. Sample Preparation

The extraction of steroid hormones and exogenous corticosteroids from serum/plasma was achieved thanks to a supported liquid extraction (SLE) protocol, employing ISOLUTE^®^ SLE+ (Biotage, Uppsala, Sweden) 400 µL 96-well plates. A total of 200 µL of each serum/plasma sample was spiked with 20 µL of the IS-mix, diluted with 200 µL of deionized water and agitated for 5 min at 600 rpm. Each well was then loaded with 400 µL of pretreated sample, and a positive pressure of 3 psi was applied for 30 s, using the Resolvex A200 (Tecan, Mannedorf, Switzerland) automated system, with the aim of facilitating sample loading and adsorption. After a waiting period of 5 min, the elution was carried out by adding 700 µL of TBME to each well and applying a pressure of 6 psi for 1 min, using the automated system. The extracts were eluted in 800 µL 96-well round collection plates, evaporated to dryness for approximately 20 min at 50 °C under a stream of air and finally reconstituted with 100 µL of a MeOH-H_2_O 1:1 (*v*/*v*) solution used as reconstitution solvent. After 10 min of shaking at 600 rpm, 20 µL of each extract was injected into the LC–MS/MS system for analyses.

### 3.3. LC–MS/MS Analysis

Analyses were performed by employing a Nexera X2 LC system (Shimadzu, Tokyo, Japan) coupled to a Citrine Triple Quad MS/MS system (AB Sciex, Ontario, Canada). System control and quantitative data analyses were carried out by AB Sciex Analyst and MultiQuant software, respectively. Liquid chromatography was performed by using an ACQUITY Premiere BEH C18 column (100 × 2.1 mm, 1.7 μm; Waters, Milford, MA, USA) equipped with an ACQUITY Premier BEH C18 VanGuard FIT Cartridge (5 × 2.1 mm, 1.7 μm; Waters, Milford, MA, USA) set at 30 °C. Mobile Phase A was 0.2 mM NH_4_F in H_2_O, and Mobile Phase B was 0.2 mM NH_4_F in MeOH. The gradient started linearly from 10% to 35% B over 0.5 min, followed by an isocratic step at 35% B for 4 min; the gradient continued with an increase from 65 to 98% B in 1.5 min, followed by a washing step at 98% B for 2 min. Finally, the column was re-equilibrated for 2 min at initial conditions for a total run time of 10 min. The injected volume was 20 μL, and the flow rate was set at 400 μL/min. ESI–MS/MS analysis was performed in positive ionization mode, with the source temperature maintained at 550 °C and ion spray voltage set at 4500 V. Curtain gas pressure was set at 35 psi, nebulizer gas pressure at 45 psi and heater gas pressure at 60 psi. Two multiple reaction monitoring (MRM) MS/MS transitions (quantifier, qualifier) were selected for each target analyte, while one MRM transition was selected for IS. The declustering potential (DP), entrance potential (EP), collision energy (CE) and cell exit potential (CXP) for each MRM transition were optimized by infusing standard solutions of each target analyte and relative IS at 100 ng/mL in reconstitution solvent. Optimized MS parameters are presented in Table 3.

### 3.4. Method Validation

The method described above was validated in compliance with the ISO/IEC requirements [39]. A validation protocol including the assessment of selectivity, extraction recoveries, matrix effects, carryover, robustness, extracts’ stability and quantitative performance (trueness, repeatability, intermediate precision, combined uncertainty, linearity range and lower limit of quantification (LLOQ)) was set up. Selectivity was assessed by extracting 5 depleted serum samples spiked with a solution containing 63 endogenous steroid hormones at concentration of 10 ng/mL and analyzing them for the evaluation of potential interferences due to circulating structurally similar compounds (the list of tested endogenous steroids is presented in Supplementary Materials Table S1). Extraction recoveries and matrix effects were calculated for each of the nine target analytes with the approach described by Matuszewski et al. [38]. Briefly, the ratio between peak areas of negative serum samples spiked before and after the extraction protocol with a mix of target analytes was used to assess extraction recoveries, while the comparison of the peak area of negative serum samples spiked after extraction with that of the corresponding methanolic standard solution containing all the target analytes was used for investigating matrix effects. For all of these tests, negative serum spiked with the Level 3 calibration solution was employed. Carryover was investigated by injecting three extracted negative controls immediately after the most concentrated calibration sample (Level 6), and the measurement of analytes’ peak areas in depleted serum samples lower than 1% of the one measured in Level 6 calibration sample was considered as proof of a negligible carryover. Lastly, quantitative validation was performed on three analytical series in three successive days, carried out by three different operators. Six calibration and six validation samples were prepared for each series in duplicate and quadruplicate, respectively, in depleted serum at appropriate concentrations for each compound (details in Supplementary Materials Table S5). Trueness, repeatability, intermediate precision, linearity range and LLOQ were determined for each analyte at each concentration level of the validation samples. Robustness was assessed by evaluating the impact of minor changes (e.g., operator performing sample extraction, mobile phase preparation, instrument maintenance and LC column lot) introduced during the three days of quantitative validation protocol, while the stability of extracted samples was investigated by analyzing external QCs and Level 5 calibration samples the day of their extraction and after storage for 3 and 7 days at 4 °C and comparing target analytes’ measured concentrations.

### 3.5. Real Samples Applications

The real samples used in the present study to test the efficiency of the validated LC–MS/MS method were collected during two different clinical protocols carried out at the City of Health and Science University Hospital of Turin. The first set of samples included serum samples collected at the Division of Endocrinology, Diabetology and Metabolism from patients enrolled in the registry of the European Network for the Study of Adrenal Tumor (ENS@T) (Prot. N. 0050191, date of approval: 14 July 2011), with suspected hypercortisolism and who underwent dexamethasone suppression test in two different dosages: 1 mg DST (1 mg Dex at 11 p.m. the day before sample collection) and LDDT (0.5 mg Dex every 6 h for two consecutive days before sample collection). Serum samples were collected in BD Vacutainer SSTTM-II Plus Advance tubes (Becton Dickinson, Franklin Lakes, NJ, USA), immediately centrifuged at 2000× *g* for 10 min and firstly analyzed with routine immunoassay of hospital’s Clinical Biochemistry laboratory employing LIAISON^®^ XL instrumentation (DiaSorin, Saluggia, Italy). Leftover serum aliquots were finally stored at −80 °C until the LC–MS/MS analysis. A total number of 64 samples coming from patients performing DST (21 males, mean age 53; 43 females, mean age 49) and 11 patients performing LDDT (2 males, mean age 67 years; 9 females, mean age 61 years) were analyzed during the present study. The second set of samples included whole-blood EDTA samples collected at the Division of Nephrology Dialysis and Renal Transplantation from patients enrolled in a study for assessing the stability of immunosuppressant drugs (Prot. N. 0097454, date of approval: 23 September 2021) and who were under treatment with Pred to prevent rejection. Whole-blood samples were collected in BD Vacutainer K2EDTA tubes (Becton Dickinson, Franklin Lakes, NJ, USA) and, following the immunosuppressant-therapeutic-drug-monitoring analysis, were centrifuged at 2000× *g* for 10 min, and plasmatic fraction aliquots of 500 μL were transferred to screw-cap polypropylene tubes (Sarstedt, Numbrecht, Germany) and stored at −80 °C until LC–MS/MS analysis. A total number of 21 plasma samples coming from transplanted patients (6 males, mean age 61 years; 15 females, mean age 54 years) were analyzed with the validated method.

## 4. Conclusions

The simultaneous measurement of F- and Dex-serum concentrations after the DST and LDDT currently represents the gold standard among clinical biochemistry tests for the diagnosis of hypercortisolism (CS and ACS). However, the implementation of rapid and sensitive MS-based analytical methods for such purpose is still not as spread in clinical context as it should be. The aim of this research work was to develop and validate a simple analytical tool for the measurement of F, E and seven exogenous corticosteroids that could be easily implemented in clinical laboratories equipped with mass spectrometry instrumentation. The presented LC–MS/MS method guarantees in a single chromatographic run of 10 min the precise and accurate measurement of selected target analytes. The encouraging performance obtained with the analysis of the real DST and LDDT samples represents a starting point for further investigations in the field of endocrinology and internal medicine that should be focused on improving the specificity of the DST and LDDT performed in routine. Indeed, it is recognized that false positives may be observed due to several individual conditions (e.g., poor Dex intestinal absorption, impaired Dex clearance and inter-individual differences in Dex metabolism), and the availability of measuring circulating Dex levels following oral administration could represent a significant step forward in routine endocrinological practice [28]. Further studies, enrolling a statistically significant population, should be performed in the future not only establish a method-specific threshold value of Dex concentration for accepting the DST and LDDT results as recommended by Keevil et al. [40], but also for investigating LC–MS/MS-specific cutoff values for the differential analysis of CS and ACS, and multicentric research works could be envisaged for introducing reference measurement procedures, as well as suitable External Quality Assessment (EQA) schemes with the aim of improving interlaboratory performance. Furthermore, the ability of the method to detect, together with F, E and Dex, also the other six exogenous corticosteroids offers clinicians a strategic investigation tool for a better understanding of non-pathological HPA-axis suppression. In this context, it would be important in the future to implement in this LC–MS/MS screening method further conventionally administered corticosteroids, such as prednisolone, methylprednisolone and fluticasone. Finally, the present study highlights once more the pivotal and emerging role of mass spectrometry in health sciences, and it should always be considered when organizing analysis workflow in clinical biochemistry laboratories and will become predominant in the next decade for therapeutic drug monitoring, as well as for specialistic markers’ measurements.

## Figures and Tables

**Table 1:** Summary of the analytical characteristics of LC–MS/MS method measuring F, E and Dex.

Reference	Matrix	Sample Volume (uL)	Sample Preparation	LC Column	Analysis Time (min)	Analytes	LLOQ (ng/mL)	Additional Analytes
Taylor RL et al. [24]	Serum Urine Table extracts	500 μL	LLE	Synergi Max_RP (50 × 4.6 mm) Phenomenex	15.00	F E Dex	0.3–0.7 0.3–0.7 0.3–0.7	13 synthetic glucocorticoids
Methlie P et al. [25]	Serum	85 μL	LLE	Zorbax RRHD C18 (50 × 2.1 mm, 1.8 μm) Agilent	6.10	F E Dex	0.7 0.6 0.03	2 synthetic glucocorticoids + 5 steroid hormones
Ceccato F et al. [26]	Serum	100 μL	PPE	Acquity UPLC HSS C18 (150 × 2.1 mm, 1.8 µm) Waters	-	Dex	0.4	-
Vogg N et al. [27]	Serum	200 μL	PPE	XBridge BEH C18 (75 × 3.0 mm, 2.5 µm) Waters	5.35	F Dex	1 1	-
Hawley JM et al. [28]	Serum	100 μL	SLE	Kinetex C8 (30 × 2.1 mm, 2.6 µm) Phenomenex	2.20	Dex	0.1	-

**Table 2:** Summary of quantitative validation results.

Compound	Ret. Time (min)	Trueness (%)	Repeatability (%)	Intermediate Precision (%)	Combined Uncertainty (%)	Linearity Range (ng/mL)	LLOQ (pg/mL)	Extraction Recovery (CV) (%)	Matrix Effect (CV) (%)
Beclomethasone	3.93	94.2–106.3	6.3–9.5	6.1–10.1	8.4–12.5	0.5–75	500	89.9 (6.3)	93.9 (0.9)
Betamethasone	3.43	98.8–105.4	5.8–8.9	6.2–9.5	9.1–13.4	0.5–60	500	85.4 (7.7)	95.5 (9.5)
Budesonide	5.77	93.6–104.9	7.1–10.3	8.3–11.1	9.9–14.0	0.5–60	500	79.5 (6.9)	87.3 (3.4)
Cortisol	2.53	97.8–105.5	5.5–8.5	5.3–9.2	8.4–12.1	1–500	1000	83.2 (6.3)	92.6 (1.5)
Cortisone	2.61	95.6–105.6	4.6–8.6	5.2–8.8	7.4–12.2	0.1–50	100	80.4 (6.5)	94.9 (0.5)
Dexamethasone	3.55	98.6–105.9	6.7–9.2	6.1–9.6	11.4–13.6	0.5–60	500	97.8 (6.2)	94.7 (9.5)
Flumethasone	3.65	93.2–108.4	6.6–9.9	7.2–10.8	8.5–13.6	1–100	1000	79.2 (4.2)	106.6 (7.8)
Prednisone	2.44	96.6–106.3	5.7–8.8	6.2–9.9	8.4–14.1	0.25–250	250	84.9 (9.7)	94.7 (0.8)
Triamcinolone	1.70	94.8–105.0	7.4–10.1	7.6–10.4	8.8–12.8	1–100	1000	67.0 (10.8)	96.6 (5.9)

**Table 3:** Optimized mass spectrometric parameters for analytes and internal standards MRM transitions. (DP, declustering potential; EP, entrance potential; CE, collision energy; CXP, cell exit potential).

Compound	Ionization Mode	Q1 Mass (Da)	Q3 Mass (Da)	DP (V)	EP (V)	CE (V)	CXP (V)
Beclomethasone	Pos	409.3 *	391.1	41	10	15	16
		409.3	147.1	41	10	23	16
Betamethasone	Pos	393.3 *	373.2	44	10	13	10
		393.3	355.2	44	10	16	10
Budesonide	Pos	413.3 ^+^	147.1	126	10	43	12
		413.3 ^+^	173.1	126	10	43	12
Budesonide d8	Pos	439.4	323.1	81	10	19	14
Cortisol	Pos	363.2 *	121.0	101	10	23	16
		363.2	90.9	106	10	83	16
Cortisol d4	Pos	367.2	121.0	106	10	31	12
Cortisone	Pos	361.2 *	163.1	121	10	28	14
		361.2	91.0	126	10	85	10
Cortisone d8	Pos	369.2	168.0	126	10	33	14
Dexamethasone	Pos	393.3 *	355.2	44	10	16	10
		393.3	373.2	44	10	13	10
Dexamethasone d3	Pos	396.3	358.2	46	10	17	10
Flumethasone	Pos	411.3 *	253.2	41	10	21	12
		411.3	121.2	41	10	63	12
Prednisone	Pos	359.3 *	171.3	135	10	46	10
		359.3	146.9	135	10	46	10
Prednisone d8	Pos	367.3	348.1	66	10	17	10
Triamcinolone	Pos	395.3 *	375.2	51	10	13	14
		395.3	165.1	51	10	95	14

* MRM transition selected for quantitation. **^+^** [M − H_2_O + H]^+^ monitored as parent ion.

## Data Availability

Not applicable.

## Supplementary Materials

The following supporting information can be downloaded at https://www.mdpi.com/article/10.3390/molecules28010248/s1. Figure S1: Extracted ion chromatograms obtained with (A) initial chromatographic conditions and (B) optimized chromatographic conditions for an extracted negative serum containing budesonide at a concentration of 10 ng/mL. Table S1: Complete list of endogenous steroid hormones included in the mixture used for testing potential interference on analytes’ selected MS transition. Table S2: Calibration line equations obtained during the three days of quantitative validation protocol for all target analytes. Table S3: Stability of extracts stored for 3 and 7 days at 4 °C after the extraction. Table S4: Summary of quantitative validation protocol parameters’ acceptance criteria and results obtained for all target analytes. Table S5: Calibration and validation samples’ composition (final concentration in serum). Table S6: Internal Standard Mix composition (final concentration in serum).

## Sample Availability

Samples of the compounds are available from the authors.
