# Supplementary material for: Simultaneous Measurement of Cortisol, Cortisone, Dexamethasone and Additional Exogenous Corticosteroids by Rapid and Sensitive LC–MS/MS Analysis
Source: Molecules. 2022 Dec 28;28(1):248. doi: 10.3390/molecules28010248 (PMC9821861; doi:10.3390/molecules28010248)
Supplement: Supplementary file 1 [file molecules-28-00248-s001.zip › molecules-2074610-supplementary.pdf]

Supplementary Material

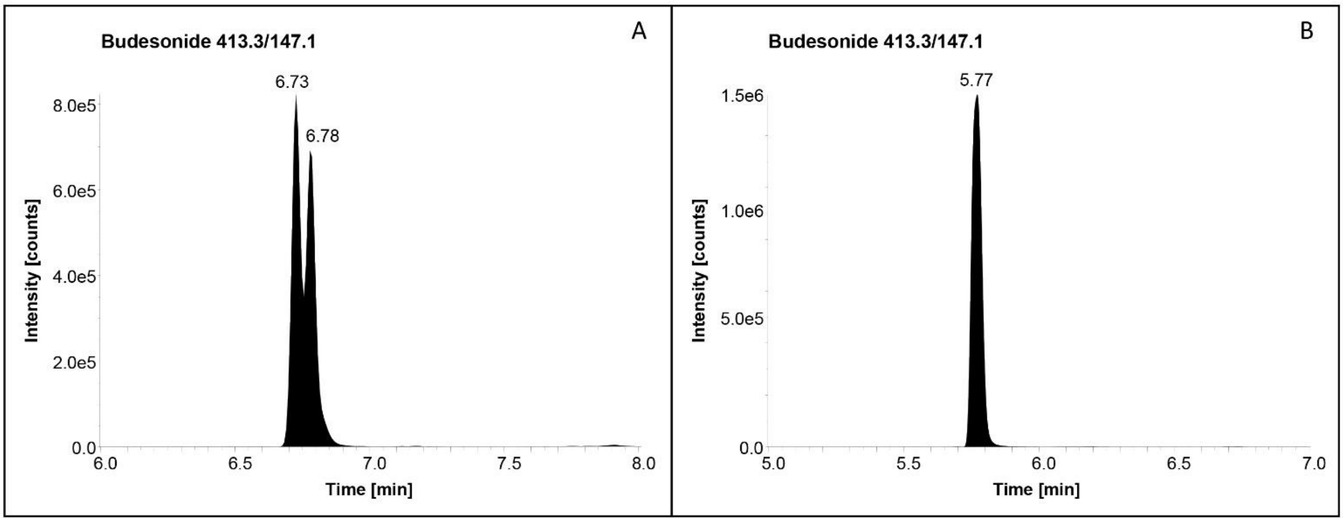

Figure S1. Extracted ion chromatograms obtained with (**A**) initial chromatographic conditions and (**B**) optimized chromatographic conditions for an extracted negative serum containing budesonide at a concentration of 10 ng/mL.

Table S1. Complete list of endogenous steroid hormones included in the mixture used for testing potential interference on analytes' selected MS transition.

Compound	Chemical Formula	Molecular Weight [Da]
11α-Hydroxyprogesterone	$C_{21}H_{30}O_3$	330.21895
11β-Hydroxyandrostenedione	$C_{19}H_{26}O_3$	302.18765
11β-Hydroxyandrosterone	$C_{19}H_{30}O_3$	306.21895
11β-Hydroxyepiandrosterone	$C_{19}H_{30}O_3$	306.21895
11β-Hydroxyetiocholanolone	$C_{19}H_{30}O_3$	306.21895
11β-Hydroxyprogesterone	$C_{21}H_{30}O_3$	330.21895
11β-Hydroxytestosterone	$C_{19}H_{28}O_3$	304.20330
11-Dehydrocorticosterone	$C_{21}H_{28}O_4$	344.19821
11-Deoxycorticosterone	$C_{21}H_{30}O_3$	330.21895
11-Deoxycortisol	$C_{21}H_{30}O_4$	346.21386
11-Ketoandrostenedione	$C_{19}H_{24}O_3$	300.17200
11-Ketoandrosterone	$C_{19}H_{28}O_3$	304.20330
11-Ketoetiocholanolone	$C_{19}H_{28}O_3$	304.20330
11-Ketotestosterone	$C_{19}H_{26}O_3$	302.18765
15β-Hydroxytestosterone	$C_{19}H_{28}O_3$	304.20330
16α-Hydroxyandrostenedione	$C_{19}H_{26}O_3$	302.18765
16α-Hydroxyandrosterone	$C_{19}H_{30}O_3$	306.21895
16α-Hydroxydehydroepiandrosterone	$C_{19}H_{28}O_3$	304.20330
16α-Hydroxyetiocholanolone	$C_{19}H_{30}O_3$	306.21895
16α-Hydroxyprogesterone	$C_{21}H_{30}O_3$	330.21895
16α-Hydroxytestosterone	$C_{19}H_{28}O_3$	304.20330
16β-Hydroxydehydroepiandrosterone	$C_{19}H_{28}O_3$	304.20330
16β-Hydroxytestosterone	$C_{19}H_{28}O_3$	304.20330

17α-Hydroxypregnenolone	$C_{21}H_{32}O_3$	332.23460
17α-Hydroxyprogesterone	$C_{21}H_{30}O_3$	330.21895
19-Hydroxyandrostenedione	$C_{19}H_{26}O_3$	302.18765
19-Hydroxytestosterone	$C_{19}H_{28}O_3$	304.20330
20α-Cortolone	$C_{21}H_{34}O_5$	366.24008
20α-Dihydrocortisone	$C_{21}H_{30}O_5$	362.20878
20α-Dihydroprogesterone	$C_{21}H_{32}O_2$	316.23968
20β-Cortolone	$C_{21}H_{34}O_5$	366.24008
21-Deoxycortisol	$C_{21}H_{30}O_4$	346.21386
21-Hydroxypregnenolone	$C_{21}H_{32}O_3$	332.23460
2β-Hydroxytestosterone	$C_{19}H_{28}O_3$	304.20330
5α-Androstane-3α,17α-diol	$C_{19}H_{32}O_2$	292.23968
5α-Androstane-3α,17β-diol	$C_{19}H_{32}O_2$	292.23968
5α-Androstane-3β,17α-diol	$C_{19}H_{32}O_2$	292.23968
5α-Androstane-3β,17β-diol	$C_{19}H_{32}O_2$	292.23968
5β-Androstane-3α,17α-diol	$C_{19}H_{32}O_2$	292.23968
5β-Androstane-3α,17β-diol	$C_{19}H_{32}O_2$	292.23968
5β-Androstane-3β,17α-diol	$C_{19}H_{32}O_2$	292.23968
5β-Androstane-3β,17β-diol	$C_{19}H_{32}O_2$	292.23968
6α-Hydroxytestosterone	$C_{19}H_{28}O_3$	304.20330
6β-Hydroxytestosterone	$C_{19}H_{28}O_3$	304.20330
7α-Hydroxydehydroepiandrosterone	$C_{19}H_{28}O_3$	304.20330
7α-Hydroxypregnenolone	$C_{21}H_{32}O_3$	332.23460
7α-Hydroxytestosterone	$C_{19}H_{28}O_3$	304.20330
7β-Hydroxydehydroepiandrosterone	$C_{19}H_{28}O_3$	304.20330
Aldosterone	$C_{21}H_{28}O_5$	360.19313
Allopregnanolone	$C_{21}H_{34}O_2$	318.25588
Allotetrahydrocortisol	$C_{21}H_{34}O_5$	366.24008
Androstenedione	$C_{19}H_{26}O_2$	286.19273
Androsterone	$C_{19}H_{30}O_2$	290.22403
Corticosterone	$C_{21}H_{30}O_4$	346.21386
Dehydroandrosterone	$C_{19}H_{28}O_2$	288.20838
Dehydroepiandrosterone	$C_{19}H_{28}O_2$	288.20838
Dihydrotestosterone	$C_{19}H_{30}O_2$	290.22403
Epitestosterone	$C_{19}H_{28}O_2$	288.20838
Etiocholanolone	$C_{19}H_{30}O_2$	290.22403
Pregnanolone	$C_{21}H_{34}O_2$	318.25533
Pregnenolone	$C_{21}H_{32}O_2$	316.23968
Progesterone	$C_{21}H_{30}O_2$	314.22403
Testosterone	$C_{19}H_{28}O_2$	288.20838
Tetrahydrocortisone	$C_{21}H_{32}O_5$	364.22443

Table S2. Calibration line equations obtained during the three days of quantitative validation protocol for all target analytes.

Compound	Calibration Line Equation		
	Day 1	Day 2	Day 3
**Beclomethasone**	y=0.16566x+0.02315 (R^2=0.99276)	y=0.16245x+0.02038 (R^2=0.99416)	y=0.15872x+0.02564 (R^2=0.99388)
**Betamethasone**	y=0.09586x+0.00067 (R^2=0.99175)	y=0.09209x+0.00065 (R^2=0.99352)	y=0.09389x+0.00061 (R^2=0.99435)
**Budesonide**	y=0.12123x+0.01405 (R^2=0.99332)	y=0.12458x+0.01722 (R^2=0.99518)	y=0.11982x+0.01900 (R^2=0.99614)
**Cortisol**	y=0.06371x+0.28312 (R^2=0.99456)	y=0.06459x+0.18898 (R^2=0.99632)	y=0.06484x+0.24131 (R^2=0.99511)
**Cortisone**	y=0.26142x-0.033084 (R^2=0.99231)	y=0.26366x-0.03790 (R^2=0.99489)	y=0.25929x-0.02819 (R^2=0.99656)
**Dexamethasone**	y=0.09079x+0.01683 (R^2=0.99721)	y=0.09241x+0.00835 (R^2=0.99374)	y=0.08818x+0.01700 (R^2=0.99456)
**Flumethasone**	y=0.04325x+0.00212 (R^2=0.99345)	y=0.04325x+0.00212 (R^2=0.99345)	y=0.08818x+0.01700 (R^2=0.99456)
**Prednisone**	y=0.00878x+0.00073 (R^2=0.99572)	y=0.00922x+0.00068 (R^2=0.99313)	y=0.00899x+0.00063 (R^2=0.99629)
**Triamcinolone**	y=0.02810x+0.00507 (R^2=0.99513)	y=0.02768x+0.00481 (R^2=0.99294)	y=0.02882x+0.00546 (R^2=0.99333)

Table S3. Stability of extracts stored for 3 and 7 days at 4 °C after the extraction.

	Sample	Concentration [ng/mL]				
		Day 0	Day 3	Recovery [%]	Day 7	Recovery [%]
**Beclomethasone**	**Val5**	28,7	28,4	99,0%	28,0	97,6%
	**Val5**	29,1	28,9	99,3%	28,4	97,6%
	**Val5**	28,9	28,3	97,9%	27,7	95,8%
	**Val5**	29,3	28,3	96,6%	27,4	93,5%
**Betamethasone**	**Val5**	25,3	24,8	98,0%	24,6	97,2%
	**Val5**	24,7	25,1	101,6%	24,8	100,4%
	**Val5**	24,3	23,9	98,4%	23,3	95,9%
	**Val5**	25,2	24,0	95,2%	22,9	90,9%
**Budesonide**	**Val5**	24,3	23,8	97,9%	23,1	95,1%
	**Val5**	23,9	24,0	100,4%	23,8	99,6%
	**Val5**	25,3	24,4	96,4%	24,0	94,9%
	**Val5**	22,9	22,7	99,1%	22,0	96,1%
**Cortisol**	**QC High**	185,2	171,3	92,5%	165,3	89,3%
	**QC High**	167,8	154,9	92,3%	155,8	92,8%
	**QC High**	170,9	165,9	97,1%	162,2	94,9%
	**QC High**	178,1	165,3	92,8%	165,5	92,9%
**Cortisone**	**QC High**	44,1	43,0	97,5%	41,6	94,3%
	**QC High**	42,8	42,1	98,4%	40,4	94,4%
	**QC High**	43,5	40,8	93,8%	41,3	94,9%
	**QC High**	42,3	41,4	97,9%	40,9	96,7%

Dexamethasone	**QC High**	45,3	44,8	98,9%	43,9	96,9%
	**QC High**	44,9	43,7	97,3%	43,0	95,8%
	**QC High**	43,7	44,0	100,7%	43,1	98,6%
	**QC High**	45,6	44,9	98,5%	44,2	96,9%
Flumethasone	**Val5**	46,2	45,0	97,4%	44,6	96,5%
	**Val5**	48,7	47,9	98,4%	48,1	98,8%
	**Val5**	51,9	50,1	96,5%	51,2	98,7%
	**Val5**	44,1	42,6	96,6%	42,9	97,3%
Prednisone	**Val5**	27,4	27,0	98,5%	26,7	97,4%
	**Val5**	28,8	28,1	97,6%	26,9	93,4%
	**Val5**	31,5	30,1	95,6%	28,5	90,5%
	**Val5**	29,9	27,4	91,6%	26,6	89,0%
Triamcinolone	**Val5**	52,7	50,3	95,4%	48,2	91,5%
	**Val5**	47,9	46,6	97,3%	45,0	93,9%
	**Val5**	50,6	51,2	101,2%	49,9	98,6%
	**Val5**	46,8	44,7	95,5%	44,2	94,4%

Table S4. Summary of quantitative validation protocol parameters' acceptance criteria and results obtained for all target analytes.

Parameter	Acceptance criteria	Beclomethasone	Betamethasone	Budesonide	Cortisol	Cortisone	Dexamethasone	Flumethasone	Prednisone	Trimacinolone
**Selectivity**	No interference (<20%LLOQ)	✓	✓	✓	✓	✓	✓	✓	✓	✓
**Linearity**	R^2>0.99	✓	✓	✓	✓	✓	✓	✓	✓	✓
**Trueness [%]**	85-115 (80-120 LLOQ)	94.2-106.3	98.8-105.4	93.6-104.9	97.8-105.5	95.6-105.6	98.6-105.9	93.2-108.4	96.6-106.3	94.8-105.0
**Repeatability [%]**	<15 (20 LLOQ)	6.3-9.5	5.8-8.9	7.1-10.3	5.5-8.5	4.6-8.6	6.7-9.2	6.6-9.9	5.7-8.8	7.4-10.1
**Intermediate Precision [%]**	<15 (20 LLOQ)	6.1-10.1	6.2-9.5	8.3-11.1	5.3-9.2	5.2-8.8	6.1-9.6	7.2-10.8	6.2-9.9	7.6-10.4
**Combined Uncertainty [%]**	<20	8.4-12.5	9.1-13.4	9.9-14.0	8.4-12.1	7.4-12.2	11.4-13.6	8.5-13.6	8.4-14.1	8.8-12.8
**LLOQ [pg/mL]**		500	500	500	1000	100	500	1000	250	1000
**Robustness**	No impact of minor modifications	✓	✓	✓	✓	✓	✓	✓	✓	✓
**Carry-over [%]**	<1 in the 1st blank afetr Cal6	0.4%	0.3%	0.3%	0.3%	0.6%	0.2%	0.4%	0.3%	0.5%
**Stability of extracts [%]**	85-115 of Day 0 value	93.5-97.6	90.9-100.4	94.9-99.6	89.3-94.9	94.3-96.7	95.8-98.6	96.5-98.8	89.0-97.4	91.5-98.6

Table S5. Calibration and validation samples composition (final concentration in serum).

Compound	Concentration [ng/mL]					
	Cal1/Val1	Cal2/Val2	Cal3/Val3	Cal4/Val4	Cal5/Val5	Cal6/Val6
**Beclomethasone**	0.5	2	7.5	15	30	75
**Betamethasone**	0.5	1.5	3	10	25	60
**Budesonide**	0.5	1.5	3	10	25	60
**Cortisol**	1	10	25	50	250	500
**Cortisone**	0.1	1	2.5	5	25	50
**Dexamethasone**	0.5	1.5	3	10	25	60
**Flumethasone**	1	5	10	25	50	100
**Prednisone**	0.25	1	2.5	10	30	75
**Triamcinolone**	1	5	10	25	50	100

Table S6. Internal Standard Mix composition (final concentration in serum).

Compound	Concentration [ng/mL]
**Budesonide d8**	10
**Cortisol d4**	15
**Cortisone d8**	5
**Dexamethasone d3**	5
**Prednisone d8**	10
